# Nucleolar PARP-1 Expression Is Decreased in Alzheimer's Disease: Consequences for Epigenetic Regulation of rDNA and Cognition

**DOI:** 10.1155/2016/8987928

**Published:** 2016-02-29

**Authors:** Jianying Zeng, Jenny Libien, Fatima Shaik, Jason Wolk, A. Iván Hernández

**Affiliations:** ^1^Department of Pathology, State University of New York, Downstate Medical Center, Brooklyn, NY 11203, USA; ^2^Department of Neurology, State University of New York, Downstate Medical Center, Brooklyn, NY 11203, USA; ^3^Department of Physics, New York University, New York, NY 10003, USA; ^4^The Robert F. Furchgott Center for Neural and Behavioral Science, State University of New York, Downstate Medical Center, Brooklyn, NY 11203, USA

## Abstract

Synaptic dysfunction is thought to play a major role in memory impairment in Alzheimer's disease (AD). PARP-1 has been identified as an epigenetic regulator of plasticity and memory. Thus, we hypothesize that PARP-1 may be altered in postmortem hippocampus of individuals with AD compared to age-matched controls without neurologic disease. We found a reduced level of PARP-1 nucleolar immunohistochemical staining in hippocampal pyramidal cells in AD. Nucleolar PARP-1 staining ranged from dispersed and less intense to entirely absent in AD compared to the distinct nucleolar localization in hippocampal pyramidal neurons in controls. In cases of AD, the percentage of hippocampal pyramidal cells with nucleoli that were positive for both PARP-1 and the nucleolar marker fibrillarin was significantly lower than in controls. PARP-1 nucleolar expression emerges as a sensitive marker of functional changes in AD and suggests a novel role for PARP-1 dysregulation in AD pathology.

## 1. Introduction

Alzheimer's disease (AD), the most common cause of dementia in the elderly, is an irreversible progressive neurodegenerative disorder clinically characterized by memory loss and cognitive decline [1]. AD is characterized pathologically by synaptic loss and by the accumulation of extracellular beta-amyloid (A*β*), neuritic plaques, and hyperphosphorylated tau in intracellular neurofibrillary tangles (NFT) [2–4]. Of these, synaptic loss most closely correlates with cognitive decline [5], whereas beta-amyloid accumulation, the presence of neuritic plaques, and NFT are the pathological markers required to make a definitive diagnosis of AD [6].

Failure of synaptic plasticity has been proposed as the mechanism underlying memory impairment in AD [7, 8]. The chromatin-remodeling enzyme poly(ADP-ribose) polymerase-1 (PARP-1) plays important roles in synaptic plasticity and memory consolidation in both* Aplysia* and rodents [9–11]. This enzyme engages in poly(ADP)-ribosylation (PAR), using nicotinamide adenine dinucleotide (NAD+) to form branched ADP-ribose polymers on nuclear acceptor proteins, such as DNA polymerases, ligases, and histones. This epigenetic modification results in the loosening of chromatin structure allowing repair proteins and transcription factors to access the DNA [12, 13]. PARP-1 activation leads to the expression of genes required for memory consolidation such as immediate early genes [14] and ribosomal RNA genes (rDNAs) in the nucleolus [15]. In addition, PARP-1 has also been shown to regulate multiple areas of nucleolar function, including the inheritance of rDNA chromatin structure, editing of precursor rRNA, and biogenesis of ribosomes in the nucleolus [16, 17]. Since synaptic plasticity has been shown to be impaired in AD, we hypothesized that this impairment may be due to a loss of PARP-1 and a disruption of PARP's role in the nucleolus in maintaining nucleolar integrity. To begin addressing this hypothesis, we compared PARP-1 expression in postmortem hippocampal brain tissue derived from patients with neuropathologically confirmed AD to control hippocampal brain tissue from patients without significant neuropathology. We show that PARP-1 positive staining of nucleoli in CA1 and CA4 hippocampal pyramidal cell neurons in AD is significantly reduced compared to controls. We suggest that memory impairment in AD may be due, in part, to this novel finding. This loss of nucleolar PARP-1 in AD appears due in part to a mislocalization of the protein from the nucleolus. Here, we present a model in which the loss of nucleolar PARP-1 precedes changes in nucleolar function and integrity seen in early stages of AD.

## 2. Materials and Methods

### 2.1. Case Material

Paraffin-embedded tissue blocks from the hippocampus were collected from deidentified archived material from the Alzheimer's Disease Research Center (ADRC) at Emory University School of Medicine, Sun Health Research Institute Brain and Body Donation Program of Sun City, Arizona [18, 19], Kings County Hospital Center, and State University of New York Downstate Medical Center.

Postmortem brain tissue was acquired from two groups of individuals (Table 1): (1) the AD group consisted of tissue from male and female patients with neuropathologically confirmed AD that meet the criteria for the diagnosis of “definite” Alzheimer's disease according to the Consortium to Establish a Registry for Alzheimer's Disease [20] and a high likelihood that dementia was due to AD by NIA Reagan criteria [21] and (2) the control group consisted of individuals, both male and female, of similar age to the AD group with no known history of dementia or neurologic disorder and without significant neuropathology. The AD cases had Braak scores of V-VI and the controls had Braak scores of 0, I, or II (Table 1).

### 2.2. Tissue Preparation

The samples were deparaffinized, hydrated, and submerged in 10 mM citrate buffer (pH 6.0) and microwave irradiated (15 min) for antigen retrieval. Then the samples were used for light or confocal microscopy as indicated by “Y” in Table 1.

### 2.3. Immunohistochemistry for PAR and PARP-1 by Light Microscopy

After antigen retrieval, slides were rinsed for 5 min with 0.1% triton X-100 in phosphate-buffered saline (PBS-Triton), treated with 3% H_2_O_2_ for 20 min, rinsed with PBS-Triton for 5 min, blocked in 2% normal horse serum in PBS-Triton for 30 min, and incubated with primary antibody (anti-PAR polyclonal, 1 : 200; Cat # 4336-BCP-100, Trevigen; and PARP-1 monoclonal antibody, 1 : 200; Cat # 1522G, AbD Serotec) overnight in a humidity chamber. The sections were then rinsed in PBS-Triton and incubated for 1 h in biotinylated secondary antibody horse anti-mouse (1 : 200) diluted in blocker (VECTASTAIN ABC systems, Vector Laboratories), rinsed again, and developed using the ABC system (Vector Laboratories, Burlingame, CA), using standard histologic procedures. For controls, sections were treated as mentioned above with omission of primary antisera (1 : 200).

### 2.4. Immunohistochemistry for PARP-1 by Confocal Microscopy

#### 2.4.1. Single Immunohistochemistry for PARP-1

For single immunofluorescent visualization, the samples were blocked for 1 h with 2% normal goat serum (NGS) in PBS-Triton and then incubated overnight with PARP-1 monoclonal antibody (1 : 200) diluted in blocker. After rinsing 3 times for 10 min each in PBS-Triton, the samples were incubated 4 h with goat anti-mouse-biotin F(ab) fragment (1 : 200) in blocker buffer, rinsed 3 times for 10 min each in PBS-Triton, and incubated for two hours with Strep Alexa 647 (1 : 200) and DAPI (1 : 500) in blocker buffer. The sections were then rinsed in PBS-Triton and in distilled water, immersed for 5 minutes in 70% ethanol containing 0.3% Sudan Black, rinsed in distilled water, and mounted on glass slides with Prolong Gold (Molecular Probes, Eugene, OR). For controls, sections were treated as mentioned above with omission of primary antisera.

#### 2.4.2. Double Immunohistochemistry for PARP-1 and Fibrillarin

The double immunohistochemistry was similar to the single immunohistochemistry except for (a) a second primary antibody (rabbit anti-fibrillarin antibody, 1 : 100; Cat # ab5821, Abcam) which was used during the incubation overnight and (b) a second secondary antibody (fluorescein goat anti-rabbit; 1 : 200 Invitrogen, Thermo Fisher Scientific, Glen Island, NY) which was used during the incubation with secondary antibodies.

### 2.5. Quantification

Qualitative assessment of the immunohistochemistry using light and confocal microscopy was performed and staining was determined to be either strong (for light microscopy) or high intensity (for confocal microscopy), weak or absent. Images were taken of each slide at a magnification of 400x and all the cells in three randomly chosen fields within the designated region were counted for presence or absence of nucleolar staining. For confocal microscopy, all images were taken at the same parameters preset on sections stained with no primary antibodies. Statistical studies using paired *t*-tests were performed.

## 3. Results

### 3.1. Loss of PARP-1 from the Nucleolus of Neurons in AD

**Figure 1:**
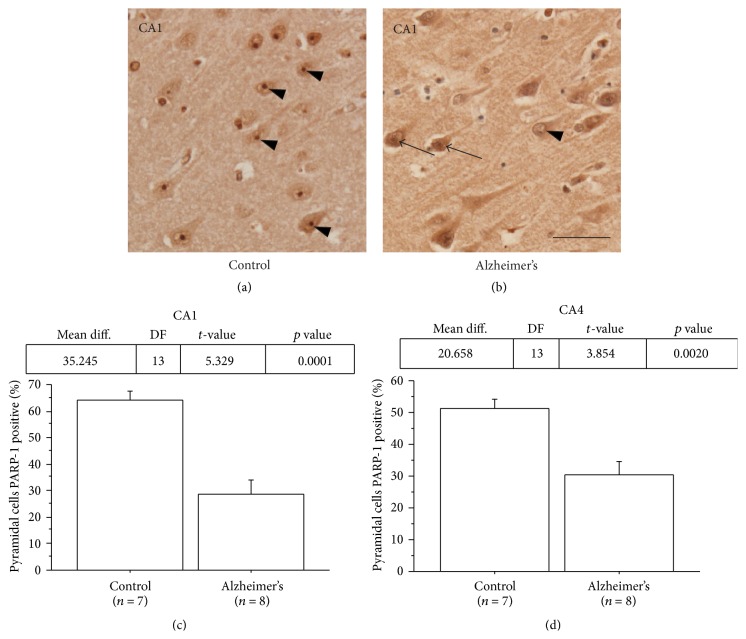
Nucleolar PARP-1 immunoreactivity in AD ranged from absent to dispersed and less intense compared to that of controls. ((a) and (b)) Representative immunostaining with diaminobenzidine (DAB) of human hippocampal pyramidal neurons in CA1 region. (a) Prominent nucleolar staining of PARP-1 (arrows) was seen in most of pyramidal neurons of a control case. (b) The nucleolar staining of PARP-1 ranged from absent (arrowheads) to a more dispersed pattern with less intensity of label (arrows) in pyramidal neurons of an AD case. ((c) and (d)) Percentages of CA1 and CA4 hippocampal pyramidal neurons with PARP-1 positive nucleoli were significantly lower in AD cases compared to controls. (Control, *n* = 8; AD, *n* = 8; ^*∗*^ *p* < 0.05.) Scale bar = 50 *μ*m.

Using light microscopy we compared PAR and PARP-1 levels in AD and controls. We found no significant differences in the nuclear staining of PAR in neurons in hippocampal regions CA1–4, entorhinal and temporal cortices, or subiculum (data not shown). In contrast, PARP-1 immunohistochemistry showed positive staining in the nucleus with strong staining of the nucleolus in controls and weak nuclear staining with little to no staining in the nucleoli within neurons in AD (Figure 1 compare (a) and (b)). Interestingly, the only exception was dentate gyrus where no differences between AD and controls were observed. In controls, the percentage of pyramidal neurons with PARP-1 positive nucleoli was 63.9% in CA1 and 51.1% in CA4. In contrast, the percentage of PARP-1 positive nucleoli in pyramidal neurons in AD was 28.7% in CA1 and 30.4% in CA4 (Figures 1(c) and 1(d)).

**Figure 2:**
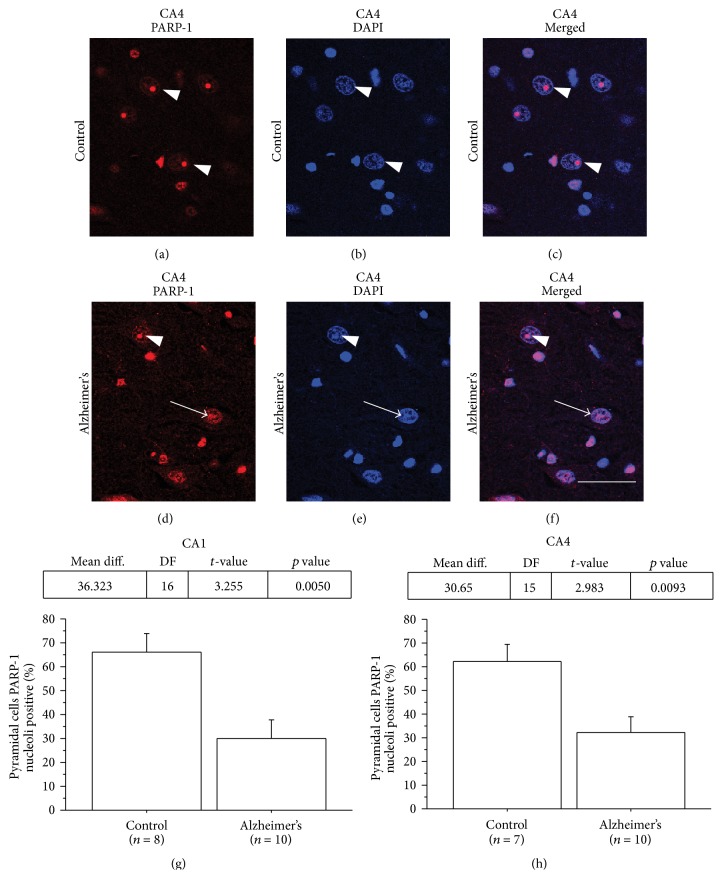
PARP-1 nucleolar immunoreactivity is altered in hippocampal pyramidal cells in AD brains. Representative confocal microscopy of PARP-1 immunostaining (red) with DAPI nuclear counterstaining (blue) of CA4 hippocampal pyramidal neurons. In controls brains (a–c) a high percentage of pyramidal cell nucleoli have intense and well- delineated PARP-1 staining (arrowheads). In contrast, in AD brains (d–f), the percentage of intensely stained and well-delineated nucleoli is less than in the controls and there is a more dispersed pattern with weak label intensity ((d) and (f), arrow). ((g) and (h)) The percentage of CA1 (g) and CA4 (h) hippocampal pyramidal neurons with PARP-1 positive nucleoli staining was less in AD cases compared to controls. (Control, *n* = 8 and *n* = 7 for CA1 and CA4, resp.; AD, *n* = 10 for both CA1 and CA4; ^*∗*^ *p* < 0.05.) Scale bar = 25 *μ*M.

We used confocal microscopy to confirm our results showing loss of PARP-1 nucleolar staining in AD. Consistent with the light microscopy data, we found that 66.1% and 62.2% of CA1 and CA4 hippocampal pyramidal cell nucleoli stained positive for PARP-1 in controls, whereas, in AD, nucleolar PARP-1 staining was present in only 29.3% and 32.0% of CA1 and CA4 pyramidal cells, respectively (Figure 2).

### 3.2. Nucleolar Marker Fibrillarin Is Not Significantly Downregulated in Nucleoli of Hippocampal Pyramidal Cells

**Figure 3:**
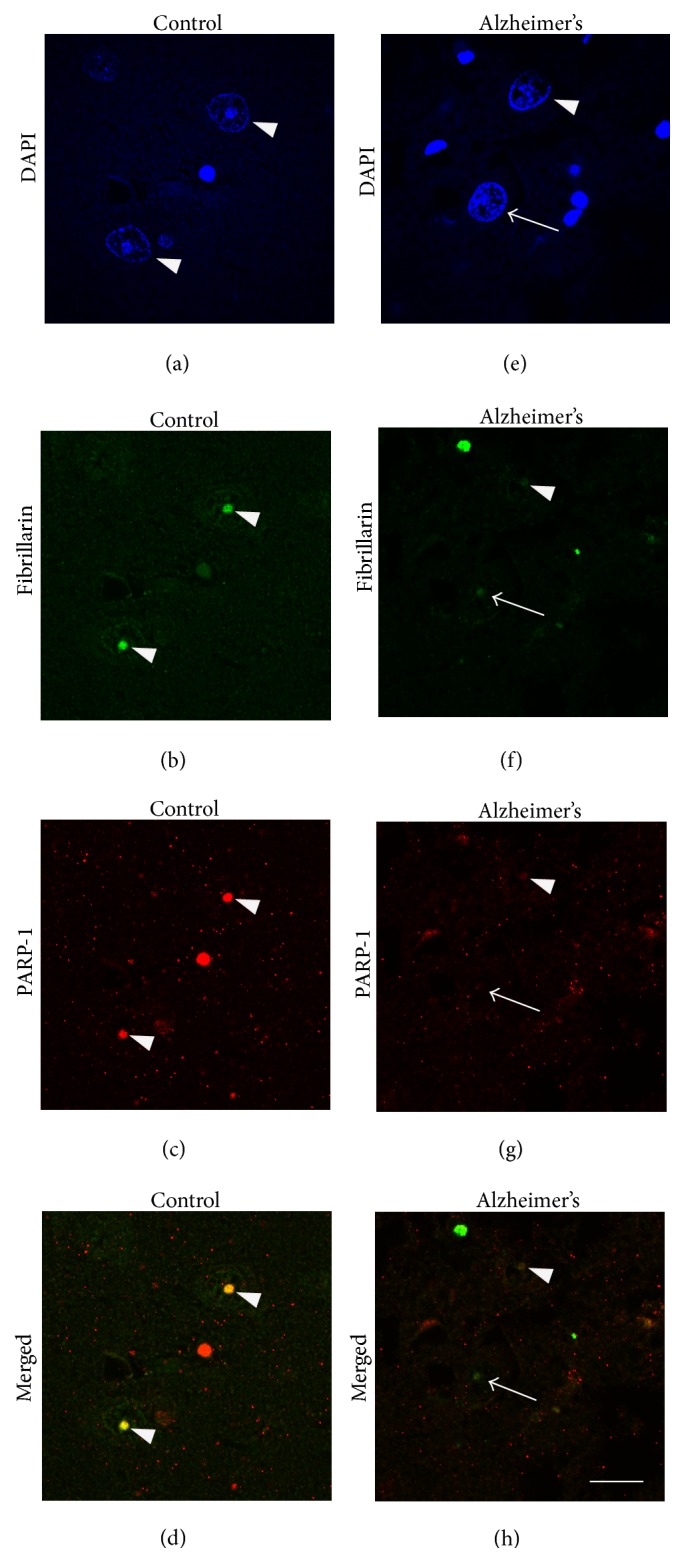
Nucleolar proteins in hippocampal pyramidal cells are altered in AD. ((a)–(h)) Representative figures show colocalization ((d) and (h), yellow) of fibrillarin ((b) and (f), green) and PARP-1 ((c) and (g), red) in the nucleoli of pyramidal neurons. Control cases exhibit high intensity staining (a–d) compared to AD (e–h) (arrowheads). In AD compared to controls, there is a lower percentage of nucleoli that are both PARP-1(+) and fibrillarin(+) ((f)-(g), arrowhead) in CA1 (see Table 2) and CA4 (see Table 3) pyramidal cells and a higher percentage of nucleoli PARP-1(−)/fibrillarin(+) ((f) and (g), arrow) in CA1 (see Table 2) and CA4 (see Table 3), suggesting that different nucleolar proteins are affected in different ways in AD. Scale bar = 20 *μ*m.

To test whether other nucleolar proteins are affected in AD, we performed double immunohistochemistry with PARP-1 and fibrillarin, a nucleolar protein involved in pre-rRNA processing. If the loss of PARP-1 nucleolar staining was due to general damage and structural loss of nucleoli from cells, then we would also expect to see a comparable loss of fibrillarin and other nucleolar proteins. However, a loss of PARP-1 with preserved fibrillarin staining in AD would indicate that loss of PARP-1 is selective. Control cases exhibited high intensity nucleolar staining and a higher percentage of PARP-1 and fibrillarin colocalization (Figures 3(a)–3(d)) compared to AD (Figures 3(e)–3(h)). There is a significant loss (*p* = 0.017) of PARP-1 nucleolar staining in CA1 pyramidal cells in AD compared to controls. In contrast, fibrillarin staining in CA1 is not significantly different between AD and controls (Tables 2 and 3). The loss of PARP-1 from the nucleolus, therefore, appears to be a selective departure and may reflect a departure from the nucleolus due to mislocalization of the protein (Tables 2 and 3).

## 4. Discussion

In this study, we demonstrated that there is a loss of PARP-1 from hippocampal pyramidal cell nucleoli in AD, suggesting that PARP-1 nucleolar function may be compromised in AD. Recently, our group demonstrated that the maintenance of late-phase long-term potentiation (L-LTP), a model for long-term memory, requires nucleolar integrity and the expression of new rRNAs—the latter being regulated by PARP-1 [22]. Therefore, we hypothesize that PARP-1 and nucleolar integrity are required for long-term memory. Recently, in a study complementary to ours, it was demonstrated that chronic deficits in nucleolar function alter synaptic plasticity and learning and memory [23]. In addition, PARP-1 has also been shown to regulate multiple areas of nucleolar function, including the inheritance of rDNA chromatin structure, editing of precursor rRNA, and biogenesis of ribosomes in the nucleolus [16, 17].

There is a previous immunohistochemical study of PARP-1 and PAR staining in AD, which found an increase in nuclear PARP-1 and PAR in frontal and temporal lobe tissues [24]. Hippocampus was not examined and the nucleolar compartment was not assessed. It is possible that the PARP-1 nucleolar loss has a differential degree of sensitivity in different areas of the brain in AD and may be a finding specific to the hippocampus. We found that the CA1 and CA4 subregions of the hippocampus exhibit vulnerability to the nucleolar PARP-1 loss in AD, which mirrors the vulnerability to AD neuropathological change and to ischemic damage. Interestingly, chronic deficits in nucleolar function have been shown to lead to neurodegeneration with differential cellular vulnerability in the hippocampus [25].

PARP-1 has shown to be activated secondary to oxidative stress and DNA damage [24, 26–28] and, in mild to moderate stress, is thought to be part of the repair mechanism but may lead to cell death via consumption of NAD+ when overactivated. We suggest that PARP-1 may act via two distinctly different mechanisms in AD. We hypothesize that the loss of PARP-1 from nucleoli of hippocampal pyramidal cells may be an early and persistent finding in AD. This loss of nucleolar PARP-1 from hippocampal pyramidal neurons may lead to deficits in synaptic plasticity and, thus, to cognitive impairment. In contrast, late in AD, it is possible that PARP-1 is overactivated and contributes to cell death in frontal and temporal cortices as shown by Love et al. [24]. We suggest that both pathways may contribute to cognitive impairment in AD. Furthermore, we speculate that the loss of PARP-1 from hippocampal pyramidal cells in AD may help to explain some of the selective vulnerability of the CA1 and CA4 regions of the hippocampus. That is, there is a loss of the physiologic PARP-1 activation required for long-term synaptic plasticity and memory consolidation [9–11, 14, 15] and also a region specific loss of the reparative activation of PARP-1 associated with mild to moderate stress.

The nucleolus has emerged as an important structure to study in relation to AD neuropathology. In a study of postmortem brains from the Nun Study of Aging and Alzheimer's Disease, a longitudinal study examining the onset of AD, it was found that asymptomatic AD cases, in which autopsied brain samples revealed common AD lesions in spite of the subjects having had normal cognition, exhibited significant hypertrophy of nucleoli (+80.2%) in CA1 neurons compared to MCI or controls [29]. There was also hypertrophy of cell bodies and nuclei but it was the nucleoli which had the largest change. This suggests a compensatory mechanism preventing the impairment of cognition despite the presence of typical AD pathology [29]. Based on these findings, we hypothesize that it was the maintenance of nucleolar function (and, therefore, rRNA synthesis) which prevented the cognitive deficits in these individuals with AD neuropathology.

Aberrations in the epigenetic code of acetylation, methylation, and PARylation are a common denominator of neurodegenerative diseases [30–32]. Nucleolar impairment may also be a common denominator in several neurodegenerative disorders such as Huntington's, Parkinson's, and Alzheimer's' disease [33]. Epigenetic silencing of rDNA by DNA methylation has been found to be a common feature of mild cognitive impairment (MCI) and AD and may represent a new marker of the disease [34]. The rDNA silencing occurs in the nucleolus, perturbing nucleolar functions such as global chromatin regulation [35] and biogenesis of ribosomes [17]. This gene silencing is consistent with previous reports of a decrease in ribosomes in the inferior parietal lobe of MCI and AD patients [36]. Impairing the expression of rRNAs (essential components of ribosomes) or any of the steps of ribosome biogenesis can produce nucleolar stress, leading to changes in gene expression and a reduction in ribosomes and protein synthesis resulting in cellular dysfunction.

**Figure 4:**
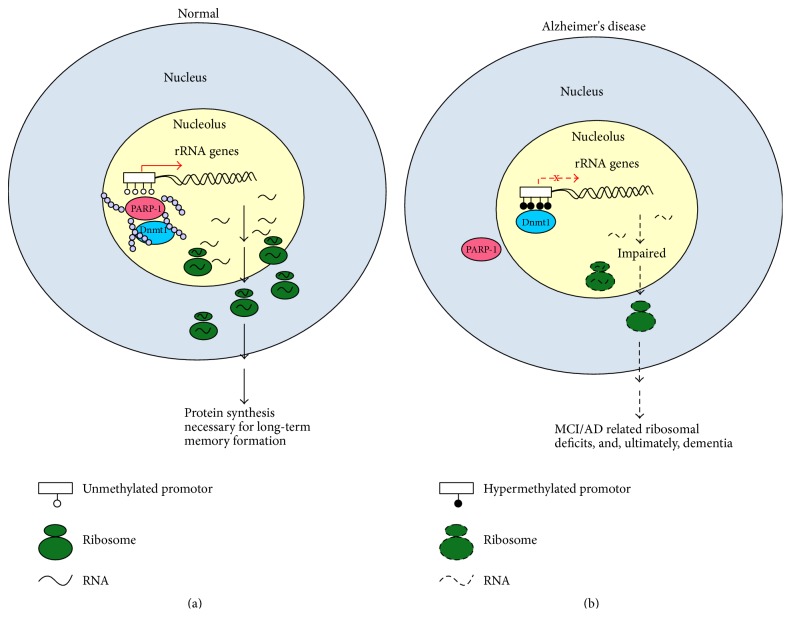
Model. Ribosome biogenesis in Alzheimer's disease. (a) Normal neuron. PARP-1 activity (PAR) prevents rDNA methylation. PAR = poly(ADPribosyl)ation. (b) AD neuron. Absence of PARP-1 in nucleoli allows DNA methyltransferase (Dnmt1) to methylate rDNA promoters silencing rRNA transcription resulting in nucleolar disruption.

To date, the factors leading to increased rDNA methylation in MCI and AD are unknown. Since PARP-1 has been shown to regulate genomic methylation patterns by inhibiting the activity of DNA methyl-transferase [37], we propose that PARP-1 displacement from the nucleolus in AD leads to hypermethylation of rDNA. There is then downregulation of rRNA expression and of ribosomal biogenesis (see Figures 4(a) and 4(b)). Without new ribosomes, the synthesis of new proteins is impaired and the formation of new memories disrupted.

## Figures and Tables

**Table 1:** Autopsy case material.

Case number	Age-sex	Diagnosis	Braak	PARP1 DAB	PARP1 confocal	PARP1/Fib confocal
1	78 F	AD	VI	Y	Y	
2	75 M	AD	VI	Y	Y	
3	77 M	AD	V	Y	Y	
4	65 F	AD	VI	Y		
5	89 F	AD	V	Y		
6	75 M	AD	V	Y		
8	87 F	AD	V	Y		
10	85 M	AD	V	Y		
11	86 M	AD	VI		Y	Y
12	76 M	AD	V		Y	Y
13	88 F	AD	V		Y	Y
14	90 M	AD	V		Y	Y
15	78 M	AD	V		Y	Y
16	65 F	AD	VI		Y	Y
17	78 F	AD	VI		Y	Y
19	81 F	Control	II	Y		
20	76 F	Control	II	Y	Y	
21	76 F	Control	II	Y		
23	69 M	Control	I	Y		
24	71 M	Control	II	Y	Y	
26	71 M	Control	II	Y		
27	71 M	Control	II	Y		
29	97 M	Control	II		Y	Y
30	93 M	Control	I		Y	Y
31	71 M	Control	I		Y	Y
32	86 M	Control	I		Y	Y
33	71 M	Control	0		Y	Y
34	44 M	Control	0		Y	Y

**Table 2:** CA1.

	% of pyramidal cells nucleoli	Mean	*t*-test
Control Alzheimer's	PARP1(+)/Fib(+)	58.80 29.74	0.039 (*∗*)

Control Alzheimer's	PARP1(+)/Fib(−)	7.17 2.53	0.329

Control Alzheimer's	PARP1(−)/Fib(+)	6.05 21.52	0.024 (*∗*)

Control Alzheimer's	Total PARP1(+)	68.60 32.27	0.017 (*∗*)

Control Alzheimer's	Total Fib(+)	64.85 51.69	0.242

^*∗*^ *p* < 0.05.

**Table 3:** CA4.

	% of pyramidal cells nucleoli	Mean	*t*-test
Control Alzheimer's	PARP1(+)/Fib(+)	55.50 26.15	0.033 (*∗*)

Control Alzheimer's	PARP1(+)/Fib(−)	5.68 4.58	0.830

Control Alzheimer's	PARP1(−)/Fib(+)	1.74 24.78	0.031 (*∗*)

Control Alzheimer's	Total PARP1(+)	61.18 30.73	0.051

Control Alzheimer's	Total Fib(+)	57.24 50.56	0.450

^*∗*^ *p* < 0.05.
